# Top Pediatric Dermatology Twitter Post Characteristics and Trends From 2020 to 2021: Content Analysis

**DOI:** 10.2196/37029

**Published:** 2022-10-26

**Authors:** Ryan E Kokoska, Lori S Kim, Mindy D Szeto, Erica L Aukerman, Robert P Dellavalle

**Affiliations:** 1 Department of Dermatology Indiana University School of Medicine Indianapolis, IN United States; 2 Department of Dermatology University of Illinois at Chicago College of Medicine Chicago, IL United States; 3 Department of Dermatology University of Colorado Anschutz Medical Campus Aurora, CO United States

**Keywords:** pediatric dermatology, pediatrics, dermatology, Twitter, social media, social media engagement, content analysis

Social media platforms, including Twitter, provide dermatologists with opportunities for collaboration [1], promotion of peer-reviewed content [2], and enhancement of disease prevention efforts [3]. However, Twitter posts (Tweets) remain largely unregulated for misinformation [1]. In previous studies, 44.7% of dermatology Twitter content was rated imprecise and 20% confusing [4]. Despite the growth of recent dermatology Twitter research, there remains a paucity of literature on pediatric dermatology Tweet content, hindering optimized information delivery. We, therefore, sought to characterize top pediatric dermatology Tweet characteristics and engagement trends in 2020 and 2021.

A search of the Twitter web application was performed periodically from August 2021 to March 2022 using the combination of hashtags #pediatrics and #dermatology, and the Twitter-designated top 3 posts for each month in 2020 and 2021 were recorded. Post content was categorized by two independent reviewers as Educational for medical information, Advertising for advertisement of a product, Promotional for promotion of an event, and Personal for all other posts, with a consensus meeting to resolve discrepancies. Posts were evaluated for Likes, Retweets, and COVID-19 content. The average Likes and Retweets for each Tweet category were tabulated and analyzed.

In total, 72 top Tweets from 2020 and 2021 were identified. Of the 72 Tweets, 43.1% (n=31) were Promotional, 36.1% (n=26) Educational, 19.4% (n=14) Advertising, and 1.4% (n=1) Personal. Two (2.7%) of the top posts were related to the COVID-19 pandemic. Promotional posts were commonly announcements for dermatology conferences, webinars, or society memberships, whereas Educational posts highlighted case reports, presentations, or publications. Overall, top posts garnered a total of 405 Likes and 101 Retweets. Compared to 2020 data, the Promotional and Educational post categories showed increased total Likes in 2021, whereas Advertising, Personal, and COVID-19 total Likes decreased (Table 1). The average number of Likes per post increased from 2020 to 2021 (5.4 to 5.9 Likes/post), with Promotional posts demonstrating the greatest increase (2.8 to 7.7 Likes/post; Table 2). Although only 1 Personal category Tweet was included, it was the most Liked (77) and Retweeted (12) post overall; it focused on the challenges faced during residency. Notably, almost half of the top Tweets were created by nonphysicians (n=35, 49%), with 31% (n=22) by physician group accounts and 21% (n=15) by single physicians.

Our results demonstrate that most pediatric dermatology top Tweets from 2020 and 2021 were Promotional and posted by roughly equal numbers of physicians and nonphysicians, with average Tweet engagement (number of Likes per post) increasing over the study interval. Additionally, we observed that Personal posts, albeit scarce, can draw significant engagement, perhaps by inspiring connection through storytelling and vulnerability [5]. Future recommendations for pediatric dermatology Twitter research include increasing the scope of hashtags chosen, analyzing other social media platforms, and examining a broader range of posts. This could expand our work and contribute to more effective patient communication and information distribution as social media engagement continues to grow.

**Table 1:** Total Likes by top pediatric dermatology Twitter post category in 2020 and 2021.

	Posts, n (%)		Likes, n (%)	
	2020 (n=36)	2021 (n=36)	2020 (n=193)	2021 (n=212)
Promotional	16 (44)	15 (42)	45 (23)	116 (55)
Educational	8 (22)	18 (50)	43 (22)	94 (44)
Advertising	11 (31)	3 (8)	28 (15)	2 (1)
Personal	1 (3)	0 (0)	77 (40)	0 (0)
COVID-19	2 (6)	0 (0)	4 (2)	0 (0)

**Table 2:** Average Likes by top pediatric dermatology Twitter post category in 2020 and 2021.

	Average Likes per post in 2020	Average Likes per post in 2021
Promotional	2.81	7.73
Educational	5.38	5.22
Advertising	2.55	0.67
Personal	77.00	0.00
COVID-19	2.00	0.00
Overall	5.36	5.89
